# Editorial: Exploration of marine microorganisms for sustainable biotechnology

**DOI:** 10.3389/fmicb.2026.1796375

**Published:** 2026-02-16

**Authors:** Jinwei Zhang, Leonel Pereira, Runying Zeng

**Affiliations:** 1 State Key Laboratory of Chemical Biology, Research Center of Chemical Kinomics, Shanghai Institute of Organic Chemistry, Chinese Academy of Sciences, Shanghai, China; 2 School of Medicine, Xiamen Cardiovascular Hospital Xiamen University, Xiamen University, Xiamen, Fujian, China; 3 Hatherly Laboratories, Faculty of Health and Life Sciences, Medical School, Institute of Biomedical and Clinical Sciences, University of Exeter, Exeter, United Kingdom; 4 Marine Resources, Conservation and Technology—Marine Algae Lab, Department of Life Sciences, CFE—Centre for Functional Ecology: Science for People & Planet, University of Coimbra, Coimbra, Portugal; 5 Engineering Innovation Center for the Development and Utilization of Marine Bioresources, Third Institute of Oceanography, Minstry of Natural Resources China, Xiamen, China

**Keywords:** biodegradation, biosynthetic pathways, environmental remediation, marine microorganisms, microbial metabolism, sustainable biotechnology

The ocean, covering over 70% of Earth's surface, harbors an estimated 10^29^ microbial cells that form the foundation of marine ecosystems and a largely untapped reservoir of biotechnological potential. As global demands for sustainable solutions intensify—from environmental remediation to novel bioproduct development—marine microorganisms have emerged as pivotal players, offering unique metabolic pathways, enzymes, and bioactive compounds shaped by millions of years of adaptation to extreme and diverse habitats. This Research Topic, “*Exploration of Marine Microorganisms for Sustainable Biotechnology*,” aims to showcase cutting-edge research that unlocks this potential while upholding the principles of ecological sustainability and responsible resource utilization.

Sustainable biotechnology relying on marine microbes addresses two critical global imperatives: harnessing nature's innovation for human needs and safeguarding the marine environment that sustains these resources. Marine microbial communities, from shallow coastal waters to the hadal zone (6,000–11,000 m depth), have evolved specialized metabolic capabilities to thrive in oligotrophic, high-pressure, low-temperature, or pollutant-rich environments. These adaptations translate into unique biocatalysts for biodegradation, novel secondary metabolites for therapeutics and cosmetics, and efficient biosynthetic pathways for industrial applications.

Yet, realizing this potential is hampered by inherent challenges. The “great plate count anomaly” leaves over 99% of marine microbes unculturable under conventional laboratory conditions, limiting direct access to their metabolic diversity. Logistical hurdles in deep-sea sampling and analysis, coupled with the need to balance resource exploitation with ecosystem preservation, further complicate progress. Fortunately, recent technological advances—including metagenomic sequencing, single-cell genomics, synthetic biology, and sustainable cultivation systems—have begun to overcome these barriers, enabling unprecedented insights into microbial diversity, function, and application that lay the groundwork for the research featured in this Research Topic.

Building on these technological breakthroughs, this Research Topic brings together five impactful publications (four research articles and one comprehensive review) that align with the core themes of marine microbial exploration, sustainable utilization, and biotechnological innovation. Collectively, these works advance our understanding of marine microbial biology while providing practical solutions for industrial and environmental challenges, directly addressing the imperatives and overcoming the barriers outlined above.

At the forefront of unlocking marine microbial potential, Han et al.'s review offers a systematic overview of fungal secondary metabolism in marine environments. It highlights genome mining as a powerful culture-independent tool to identify cryptic biosynthetic gene clusters (BGCs) in marine fungi—many of which remain silent under standard laboratory conditions—directly addressing the challenge of accessing unculturable microbes' metabolic diversity. The review synthesizes strategies for activating these BGCs—including epigenetic modification, co-cultivation, and heterologous expression—and emphasizes the sustainability of targeted metabolite discovery over traditional random screening. By cataloging diverse bioactive compounds (e.g., polyketides, non-ribosomal peptides) and their biosynthetic pathways, this work provides a roadmap for sustainable development of marine fungal resources in medicine and cosmetics, translating microbial adaptations into tangible biotechnological applications.

Complementing the review's focus on metabolite discovery, Wen et al.'s research zeroes in on thraustochytrids, a group of marine protists renowned for omega-3 polyunsaturated fatty acid (PUFA) production—key compounds with broad applications in food, pharmaceuticals, and nutraceuticals. Through comparative genomics and phylogenomic analysis, the study reveals mechanisms of ecotype divergence in thraustochytrids from different marine habitats (e.g., coastal sediments vs. open ocean), shedding light on how environmental adaptation drives metabolic specialization. Key findings include habitat-specific gene expansion related to lipid biosynthesis and environmental adaptation, providing a genetic basis for selecting strains with high PUFA yields. This research advances sustainable large-scale production of microbial lipids, offering an eco-friendly alternative to traditional fish oil extraction and reducing pressure on marine fisheries—directly contributing to the dual imperative of harnessing microbial innovation while protecting marine ecosystems.

Taking a targeted biotechnological approach, Yuan et al.'s study demonstrates innovative metabolic engineering of a marine-associated fungus, building on the BGC activation strategies highlighted in Han et al.'s review. The researchers identified phoE, a polyketide synthase gene, as a negative regulator of monoterpenes biosynthesis in Diaporthe sp. SYSU-MS4722 (isolated from an ascidian). By deleting phoE, they achieved significant induction of monoterpenes—high-value compounds in cosmetics, fragrances, and pharmaceuticals. This work exemplifies sustainable bioprocess development by unlocking novel metabolite production in a marine-derived fungus, avoiding reliance on chemical synthesis and reducing environmental footprint, which aligns with the Research Topic's focus on translating microbial potential into sustainable products.

Addressing one of the most pressing global challenges—marine pollution—two research articles in this Research Topic leverage marine microbes' biodegradative adaptations to tackle persistent pollutants. Wang et al. reports the discovery of a novel Devosia species from the Kermadec Trench (a deep-sea environment with natural PAH exposure) with exceptional capacity to degrade high-molecular-weight (HMW) PAHs—persistent organic pollutants (POPs) with severe ecological and human health impacts. The strain exhibits robust degradation efficiency under low-temperature and high-pressure conditions, mirroring its native habitat, showcasing how microbial adaptations to extreme environments can be harnessed for bioremediation.

Complementing this work, Sardar identifies a marine Metabacillus niabensis strain capable of degrading polyethylene (PE)—one of the most abundant and recalcitrant plastics in marine environments. The study characterizes the biodegradation pathway and key enzymes involved, providing evidence for microbial adaptation to plastic pollution in marine ecosystems. Together, these two studies align with emerging research highlighting marine microbes as critical agents for environmental self-repair, offering practical strains and mechanisms for developing sustainable bioremediation technologies that address the imperative of safeguarding the marine environment while solving human-induced pollution challenges.

The publications in this Research Topic represent significant strides in marine microbial sustainable biotechnology, but several frontiers remain to be explored to fully realize the potential outlined in the thematic landscape. First, advancing sustainable cultivation techniques for unculturable microbes—such as microfluidic-based single-cell cultivation and in situ simulation of marine habitats—will expand access to microbial diversity beyond what current culture-independent methods can achieve. Second, integrating synthetic biology with genome mining (as demonstrated in Yuan et al.'s metabolic engineering work) will accelerate the design of microbial cell factories for scalable production of high-value compounds, addressing the industrial application gap. Third, conducting comprehensive life cycle assessments (LCAs) of marine microbial bioprocesses—from sampling to production—is essential to quantify environmental impacts and ensure true sustainability, reinforcing the dual imperative of harnessing innovation and protecting ecosystems. Finally, addressing ethical and regulatory considerations for marine bioprospecting will safeguard marine ecosystems while promoting innovation, ensuring that future exploration adheres to responsible resource utilization principles.

